# Biomechanical effect of different plate-to-disc distance on surgical and adjacent segment in anterior cervical discectomy and fusion - a finite element analysis

**DOI:** 10.1186/s12891-021-04218-4

**Published:** 2021-04-09

**Authors:** Xing Guo, Jiaming Zhou, Yueyang Tian, Liang Kang, Yuan Xue

**Affiliations:** 1 Department of Orthopaedic Surgery, Tianjin Medical University General Hospital, Heping District, Tianjin, 300052 China; 2 Tianjin Key Laboratory of Spine and Spinal Cord, Tianjin Medical University General Hospital, Tianjin, 300052 China

**Keywords:** Finite element analysis, Plate-to-disc distance, Biomechanical effect, Adjacent segment degeneration, Spinal fusion, Degenerative disc disease, Cervical vertebrae, Spine surgery, Complication

## Abstract

**Background:**

The plate-to-disc distance (PDD) is an important factor affecting the degeneration of adjacent segments after anterior cervical discectomy and fusion (ACDF). However, the most suitable PDD is controversial. This study examined the adjacent intervertebral disc stress, bone graft stress, titanium plate stress and screw stress to evaluate the biomechanical effect of different PDD on surgical segment and adjacent segment following C5/C6 ACDF.

**Methods:**

We constructed 10 preoperative finite element models of intact C4–C7 segments and validated them in the present study. We simulated ACDF surgery based on the 10 intact models in software. We designed three different distance of plate-to-disc titanium plates: long PDD (10 mm), medium PDD (5 mm) and short PDD (0 mm). The changes in C4/C5 and C6/C7 intervertebral disc stress, bone graft stress, titanium plate stress and screw stress were analyzed.

**Results:**

The von Mises stress of C4/C5 and C6/C7 intervertebral discs had no significant differences (*P* > 0.05) in three different PDD groups. Titanium plate stress increased as the PDD decreased. The bone graft stress and screws stress decreased as the PDD decreased. The maximum stress of each part occurred was mostly in the conditions of rotation and lateral bending.

**Conclusions:**

The PDD has no effect on adjacent intervertebral disc stress, but it is an important factor that affecting the bone graft stress, titanium plate stress and screws stress after ACDF. Shorter PDD plate can provide better stability to reduce stress on screws and bone graft, which may be helpful to prevent cage subsidence, pseudarthrosis and instrument failure. This can serve as a reference for clinical choice of plate.

## Background

Cervical spondylosis is an age-related condition of the cervical spine resulting from progressive intervertebral disc degeneration. Approximately 80 to 90% of people have disc degeneration on magnetic resonance imaging by the age of 50 years [1, 2]. Disc degeneration can potentially cause compression of the nerve roots or spinal cord, resulting in radiculopathy (annual incidence of approximately 83/100000) or myelopathy (annual incidence of approximately 4/100000) [3–7]. This can manifest as a range of symptoms, including axial neck pain, radicular pain, motor weakness and sensory loss [1, 4]. For patients unresponsive to appropriate nonsurgical measures for at least 6 months, surgical treatment should be considered [8].

Anterior cervical discectomy and fusion (ACDF) is considered a standard surgical treatment for affected patients in whom nonsurgical treatments fail [9–11]. With the development of surgical instruments and surgical techniques, titanium plate has been routinely used in ACDF surgery. Reasonable use of titanium plate can provide immediate stability in early postoperative period, which can prevent bone graft subsidence, bone graft extrusion and improve fusion rates, and reduce the need for external immobilization [12, 13]. However, it has been reported that the cervical arthrodesis with plate can change the biomechanical environment of cervical spine, eventually result in adjacent segment degeneration (ASD) [14–16].

ASD is defined as the degeneration of adjacent level of spine arthrodesis. Previous studies have been reported that the repeat surgery rate of ACDF patients was 17.4% due to symptomatic ASD [17], which seriously affected the patient’s satisfaction with the surgery and increased the economic burden on society. There have been studies suggested that plate-to-disc distance (PDD) [14], graft type [18], and post-operative cervical alignment [19] are factors that affect the incidence of ASD. Among them, PDD is considered to be an important but controversial factor affecting the development of ASD. Chung et al. [14] found most clinical adjacent-segment degeneration appeared on the patients with a PDD less than 5 mm. Hence, they concluded that the PDD should be 5 mm or more if possible for preventing ASD. Yu et al. [20] found that PDD < 5 mm is a risk factor for ASD through logistic regression analysis. However, Yang et al. [21] considered that there is no correlation between PDD and the incidence of ASD. Hence, the appropriate PDD in ACDF still need to studied.

Finite element analysis (FEA) has the ability to simulate a variety of complex body structures in a computer and calculate the pressure and stress of each component without any invasion [22, 23]. FEA also provide time- and cost- effective means to address various what if scenarios, thereby reducing the need for costly experimental animal and cadaveric studies. Untill now, the biomechanical effect of different PDD on adjacent and surgical segments has not been reported through FEA. Hence, the objective of the present study is to analyze the biomechanical effect of different PDD on adjacent intervertebral disc stress, bone graft stress, titanium plate stress and screw stress precisely using quantitative FEA.

## Methods

### Construction of the C4-C7 finite element models

Ethical approval of this study was granted by the Institutional Review Board of Tianjin Medical University General Hospital (Tianjin, China). Twenty healthy volunteers were recruited from Tianjin Medical University and imaging data were obtained. The radiological data of these volunteers were reviewed by a senior doctor. Four participants with cervical degeneration or obvious natural variability were excluded. Finally, we randomly selected 10 participants and built 10 three-dimensional finite element (FE) models based on computed tomography (CT) scans with interval 0.625 mm of the cervical spine of these 10 healthy volunteers.

We used the threshold segmentation, region grow, etc. of the MIMICS 21.0 (Materialise, Leuven, Belgium) software to establish the primary geometric structures of the C4-C7 cervical vertebraes based on the CT images. Then, a smoothing process was performed in Geomagic Studio 14.0 (3D Systems, Rock Hill, SC, USA) to remove spikes and holes on the surface of the vertebral geometries. The 10 smooth models were processed using sketch, extrude, loft, cut, chamfer, etc. of Solidworks 2019 (Dassault Systèmes, Paris, France) to construct structures such as intervertebral discs, ground substance, nucleus pulposus, endplates, cartilages of the articular processes. Then geometric models were imported into Hypermesh 14.0 (Altair, Troy, MI, USA) to construct annulus fibers and ligaments, and mesh. The intervertebral disc was divided into hexahedral meshes. We constructed the annulus fibers by connecting the vertices of the hexahedrons, so the direction and angle of the annulus fibers could be controlled through adjusting the length of each side. At this point, the three-dimensional finite element models have been created. Finally, the boundary conditions and loading conditions of the prepared models were set using ABAQUS 6.9.1 (Dassault Systèmes, Paris, France).

The bony structures of the vertebral body include cortical bone, cancellous bone and posterior structure. The cancellous bone region of the vertebrae was set as solid element. The thick of the cortical bone was 1.5 mm. We created the nucleus pulposus and annulus fibrosus with a volume ratio of 4:6 [24]. Annulus fibers surrounded the ground substance with an inclination to the transverse plane between 15° and 45°, accounting for approximately 19% of the entire annulus fibrosus volume [24]. A bond connection was defined between the face-to-face contact except the cartilages of the articular processes. The cartilages were inserted into the spaces of the bony articular process joints. All cartilages of the articular processes were subjected to a face-to-face frictionless contact with each other [24, 25]. Five groups of ligaments, including the anterior longitudinal ligament (ALL), posterior longitudinal ligament (PLL), ligamentum flavum, interspinous ligament and capsular ligament were established using tension-only spring elements and attached to the corresponding vertebrae.

**Fig. 1:**
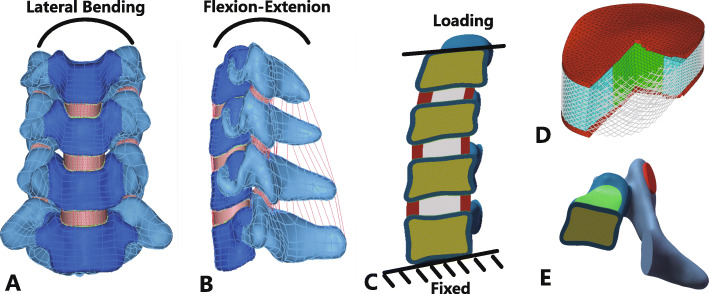
Finite element models of C4–C7 cervical spine and other structures and details. **a** Frontal view of preoperative model; **b** Lateral view of preoperative model; **c** Loading and boundary condition; **d** Intervertebral disc details; **e** Vertebral body details

The preoperative C4-C7 FE models and structural details were shown in Fig. 1. The information of ten volunteers was shown in Table 1. The material properties of the FE models are listed in Table 2 and Table 3 [26, 27].

**Table 1:** Demographics of study participants (*n* = 10)

Variables	Median (Q1, Q3)	Range
Age (years)	26.00 (24.75,27.25)	(23.00–29.00)
Height (cm)	169.50 (162.25,173.50)	(160.00–177.00)
Weight (kg)	63.75 (56.88,73.13)	(54.00–80.00)
BMI (kg/m^2^)	22.30 (21.51,24.49)	(21.09–25.54)

*BMI* Body mass index

**Table 2:** The material properties of the spinal soft tissues and hard tissues used in the finite element model

Description	Element Type	Young’s Modulus (MPa)	Poisson’s Ratio
Cortical bone	3-noded triangular shell	12,000	0.3
Cancellous bone	4-noded tetrahedron	100	0.2
Posterior elements	4-noded tetrahedron	3500	0.25
Facet cartilage	4-noded tetrahedron	10.4	0.4
End plate	4-noded tetrahedron	600	0.3
Nucleus pulposus	8-noded brick	1	0.49
Annulus ground substance	8-noded brick	3.4	0.4
Annulus fibers	Truss (tension-only)	450	0.45
Titanium plate	4-noded tetrahedron	120,000	0.3
Titanium screw	4-noded tetrahedron	120,000	0.3

**Table 3:** The material properties of the ligaments

ALL		PLL		LF		ISL		CL	
Displacement (mm)	Force (N)	Displacement (mm)	Force (N)	Displacement (mm)	Force (N)	Displacement (mm)	Force (N)	Displacement (mm)	Force (N)
0	0	0	0	0	0	0	0	0	0
1	35.5	0.9	1.33	1.7	2.2	1.2	0.75	1.7	2.452
2	64.9	2	29.0	3.74	45.9	2.7	16.9	3.9	53.6
4	89.7	3	51.4	5.61	82.9	4.0	24.4	5.8	87.9
5	108.6	4	71.38	7.48	119.6	5.4	29.5	7.7	109.4
6	119.6	5	85.8	9.35	133.7	6.7	32.9	9.7	125.8
		6	94.7	11.3	147.2	8.1	34.9	11.5	134.8

*ALL* Anterior longitudinal ligament, *CL* Capsular ligament, *ISL* Interspinous ligament, *LF* Ligamentum flavum, *PLL* Posterior longitudinal ligament

### Boundary and loading conditions

The bottom endplate of the C7 vertebral body was constrained in all degrees of freedom. The loading process involved two steps. The 73.6 N of axial compression loading were applied at the superior endplate of the C4 vertebral body that represented the head weight. After the FE models stabilized from the axial compression loading, the state was saved and used as an initial condition for the second step. In second step, the conditions loading with 1.0 N·m torque were tested for cervical spine movement directions: flexion, extension, lateral bending, and axial rotation, respectively. The loading conditions were also applied at the superior endplate of the C4 vertebral body. The values of axial compression loading and the torque were obtained from previous published studies [28, 29].

### Mesh convergence test

One of the ten FE models (Volunteer 1) was tested for mesh convergence. Three mesh resolutions were generated consecutively (in the order of Mesh 1, Mesh 2, and Mesh 3) for this FE model. Mesh 1 had the smallest number of elements and nodes among the three mesh resolutions. Mesh 2 and Mesh 3 had approximately doubled numbers of elements and nodes than the previous mesh resolution. The number of elements and nodes for each mesh resolution are shown in Table 4. The three mesh resolutions were tested under the same rotation with a moment of 1.0 N·m. The von Mises stress was calculated and compared for different structures in the FE model. When the prediction results obtained by two consecutive mesh resolutions have differences smaller than 5%, the mesh was considered to be convergent [30, 31].

**Table 4:** Element and node numbers for three different mesh resolutions

	Element number	Node number
Mesh 1	62,340	146,282
Mesh 2	129,770	282,016
Mesh 3	186,736	396,958

### Validation of the model

The range of motion (ROM) of the C4-C7 FE models were predicted with a pure bending moment of 1.0 N·m for flexion, extension, axial rotation, and lateral bending with 73.6 N of axial compression superior to C4, and compared to previous experimental results [32]. If the predicted data were within the standard deviation of the previous literature, the FE model was considered to be validated. To measure the ROM, we established a cross coordinate system on the superior plane of the target vertebral body, and then measured the ROM in different directions according to the changes in the position of the coordinate system after loading.

### Surgery simulation

**Fig. 2:**
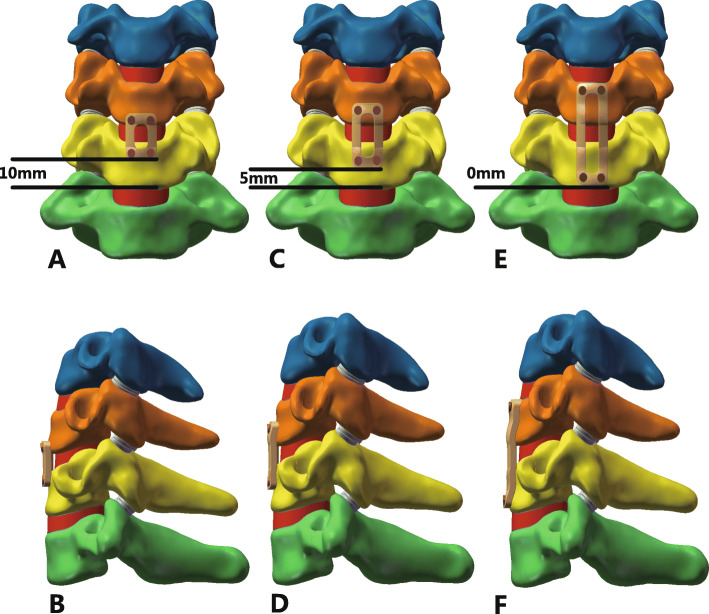
**a** and **b** Frontal and lateral view of postoperative model with long PDD titanium plate; **c** and **d** Frontal and lateral view of postoperative model with middle PDD titanium plate; **e** and **f** Frontal and lateral view of postoperative model with short PDD titanium plate. *PDD* plate-to-disc distance

During the actual surgery, the C5/C6 anterior longitudinal ligament, C5/C6 disc, inferior endplate of C5, superior endplate of C6 and C5/C6 posterior longitudinal ligament were resected. We deleted the corresponding structures to simulate the surgery more precisely. The bone graft was assumed bound to the adjacent vertebral body completely. In the present study, three different PDD plates: 0 mm, 5 mm, 10 mm plates and self-tapping screws were simulated. The screws were fixed in parallel to the endplates in all postoperative models. The 10 postoperative models was loaded in flexion, extension, axial rotation, and lateral bending by imposing a pure moment of 1.0 N·m on C4 with 73.6 N of axial precompression superior to the upper endplate of C4. The lower endplate of C7 was firmly fixed in all degrees of freedom. The postoperative C4-C7 FE models with different PDD plates were shown in Fig. 2. We choose the von Mises stress of adjacent intervertebral disc, the titanium plate, bone graft and screws as the parameters to evaluate mechanical effect of three different PDD plates.

### Statistical analysis

In order to investigate similarities and differences for the von Mises stress of adjacent intervertebral disc, the titanium plate, bone graft and screws among the three groups under flexion, extension, axial rotation, and lateral bending loading conditions, we used multivariate analysis of variance (MANOVA). Before MANOVA, Shapiro-Wilk test, colinearity diagnostics and Box’s test were conducted. All data of the three groups were greater than 0.05 in Shapiro-Wilk test, indicating that the data were normally distributed. In colinearity diagnostics, all variance inflation factors were less than 10 and tolerances were greater than 0.1, meaning that no significant multicollinearity existed among the variables. The *p* values in Box’s test were greater than 0.05, demonstrating the covariance matrices were equal. Then, the MANOVA was conducted. For post hoc test, Tukey method (equal variance assumed) or Games-Howell method (equal variance not assumed) was used. A *p* value of less than 0.05 was considered significant. Results are presented as mean ± standard deviation. All statistical analysis was done with SPSS Version 25.0 (IBM, Armonk, NY, USA).

## Results

### Characteristics of volunteers

The characteristics of the 10 volunteers are shown in Table 1. The median age of the volunteers is 26 years, ranged from 23 to 29 years. The median height, weight and BMI were 169.50 cm, 63.75 kg and 22.30 kg/m^2^, respectively.

### Mesh convergence test

**Fig. 3:**
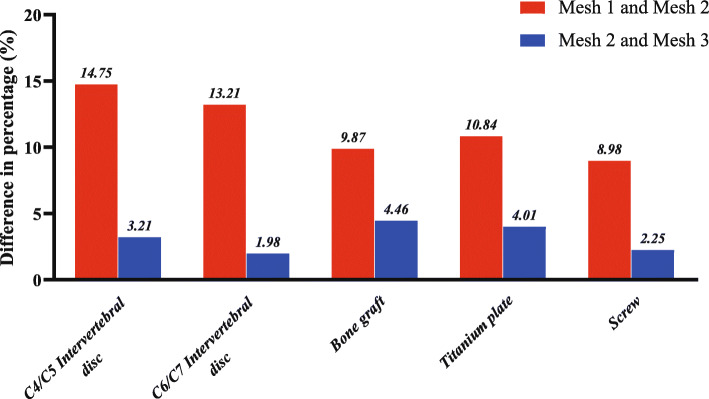
The predicted percentage differences of von Mises stress between Mesh 1 and Mesh 2 and between Mesh 2 and Mesh 3 in different structures for Volunteer 1 in the axial rotation

The percentage differences in von Mises stress of Mesh 1 vs. Mesh 2 and Mesh 2 vs. Mesh 3 are shown in Fig. 3. The differences of von Mises stress between Mesh 2 and Mesh 3 were less than 5% in the model. Hence, Mesh 2 was considered to be stress-converged. All the 10 FE models were meshed in stress converged mesh resolution (Mesh 2 level).

### FE model validation

**Fig. 4:**
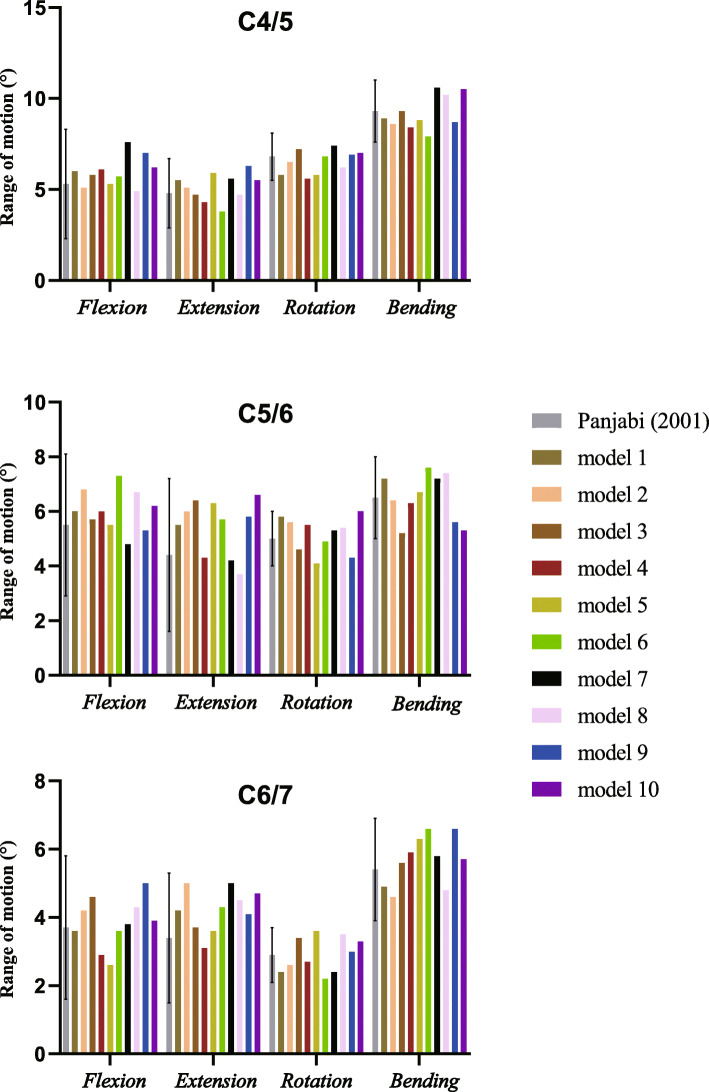
The predicted ROM of the preoperative model was validated by previous published study. All the predicted data in our study occurred within the standard deviation of the mean values of the previous literature. *ROM* range of motion

The comparisons between in vitro data and predicted values in the FE models are shown in Table 5 and Fig. 4. All the predicted data in this study occurred within the standard deviation of the mean values of the previous literature [32], meaning the data was in a good agreement with published experimental results. Hence, the FE models can be regarded as validated and could be used in the present study.

**Table 5:** ROM (°) of each segment under different loading conditions

Load scheme	Experiment data (mean ± SD)			Current model [median (min, max)]		
	C4/5	C5/6	C6/7	C4/5	C5/6	C6/7
Flexion	5.3 ± 3.0	5.5 ± 2.6	3.7 ± 2.1	5.9 (4.9, 7.6)	6.0 (4.8, 7.3)	3.9 (2.6, 5.0)
Extension	4.8 ± 1.9	4.4 ± 2.8	3.4 ± 1.9	5.3 (3.8, 6.3)	5.8 (3.7, 6.6)	4.3 (3.1, 5.0)
Rotation	6.8 ± 1.3	5.0 ± 1.0	2.9 ± 0.8	6.7 (5.6, 7.4)	5.4 (4.1, 6.0)	2.9 (2.2, 3.6)
Lateral bending	9.3 ± 1.7	6.5 ± 1.5	5.4 ± 1.5	8.9 (7.9, 10.6)	6.6 (5.2, 7.6)	5.8 (4.6, 6.6)

*ROM* Range of motion, *SD* Standard deviation

### The von Mises stress in the different structures

**Fig. 5:**
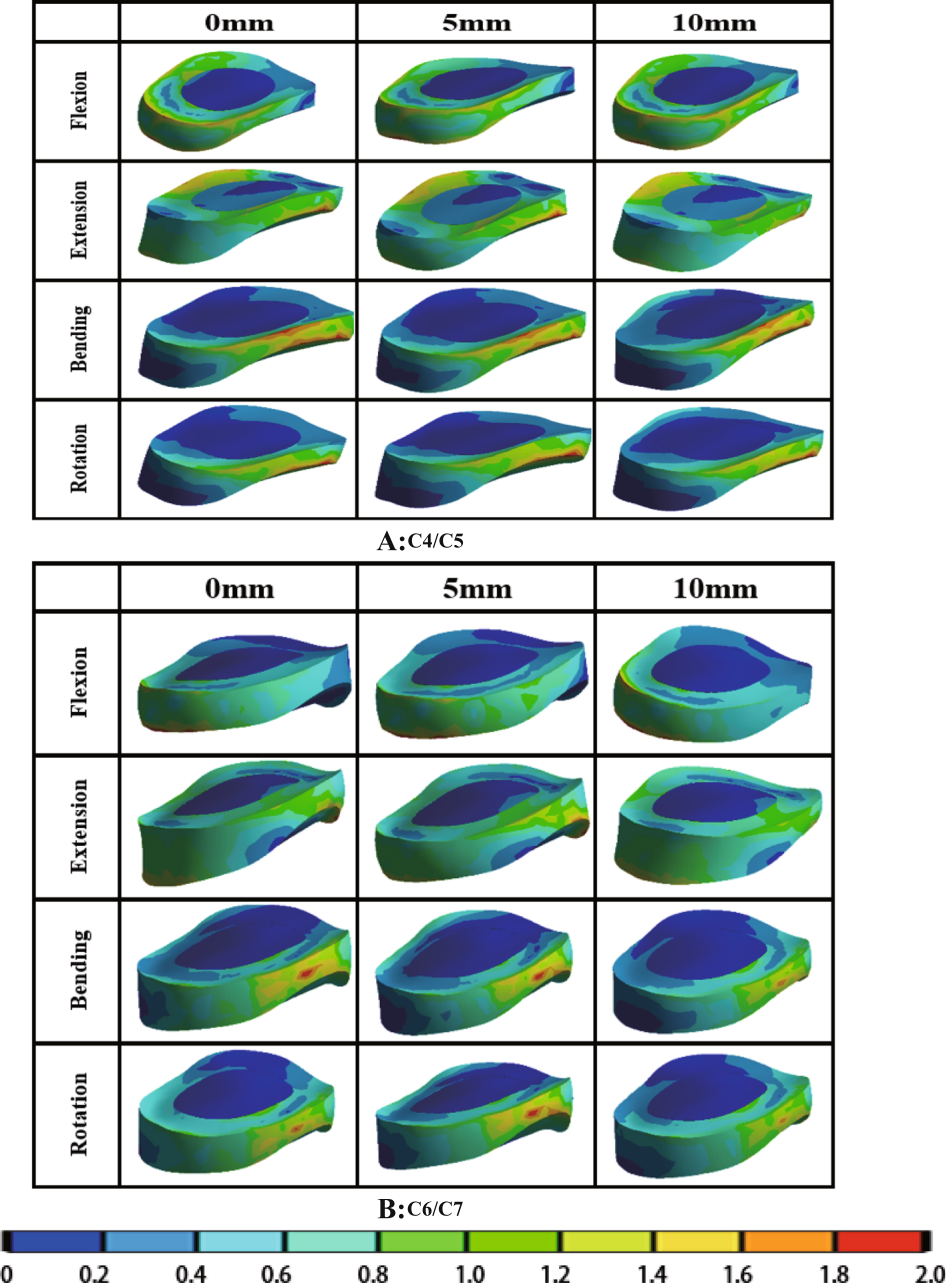
The von Mises stress distribution diagram of adjacent intervertebral discs with different PDD titanium plates under different loading conditions (**a** C4/C5; **b** C6/C7). *PDD* plate-to-disc distance

**Fig. 6:**
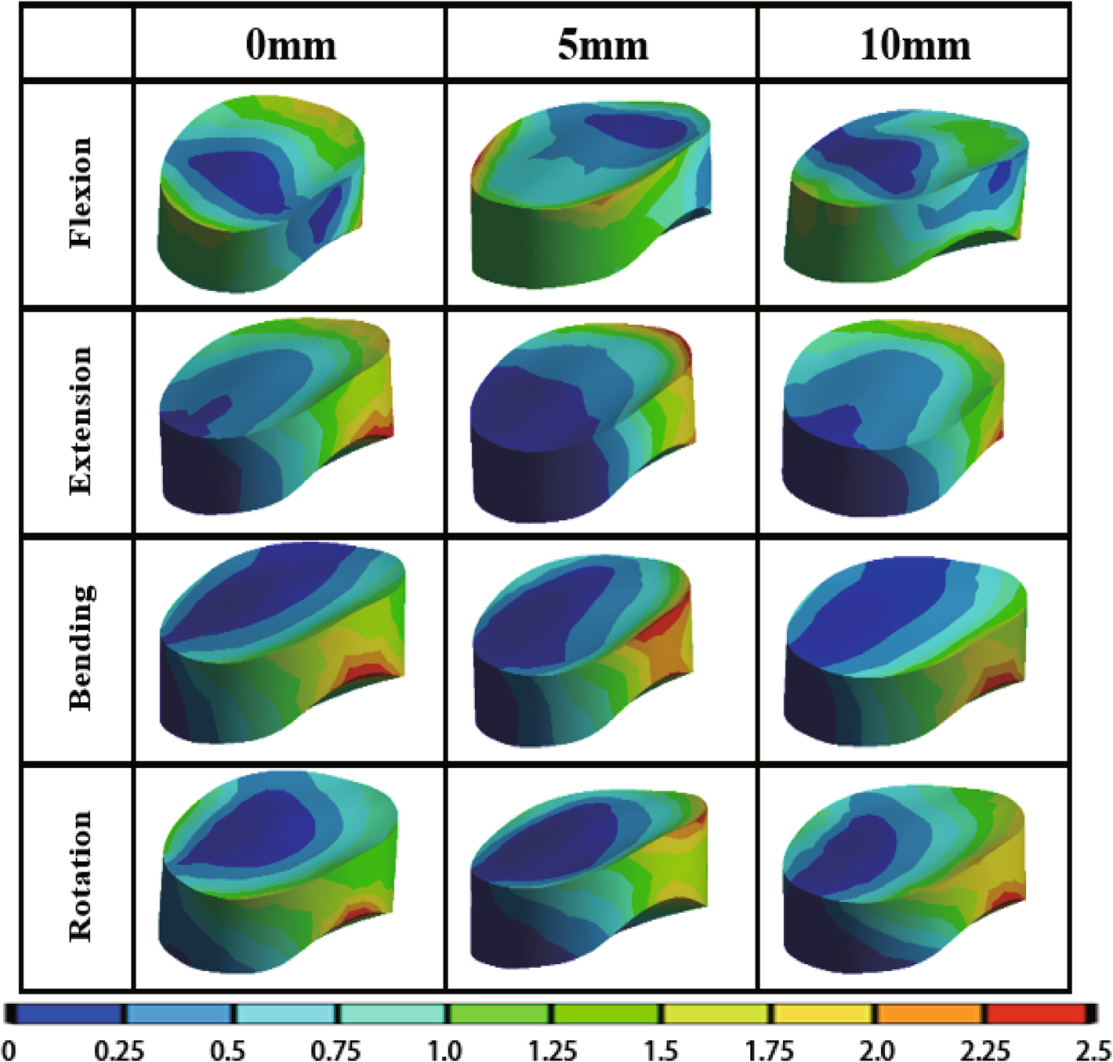
The von Mises stress distribution diagram of bone graft with different PDD titanium plates under different loading conditions. *PDD* plate-to-disc distance

**Fig. 7:**
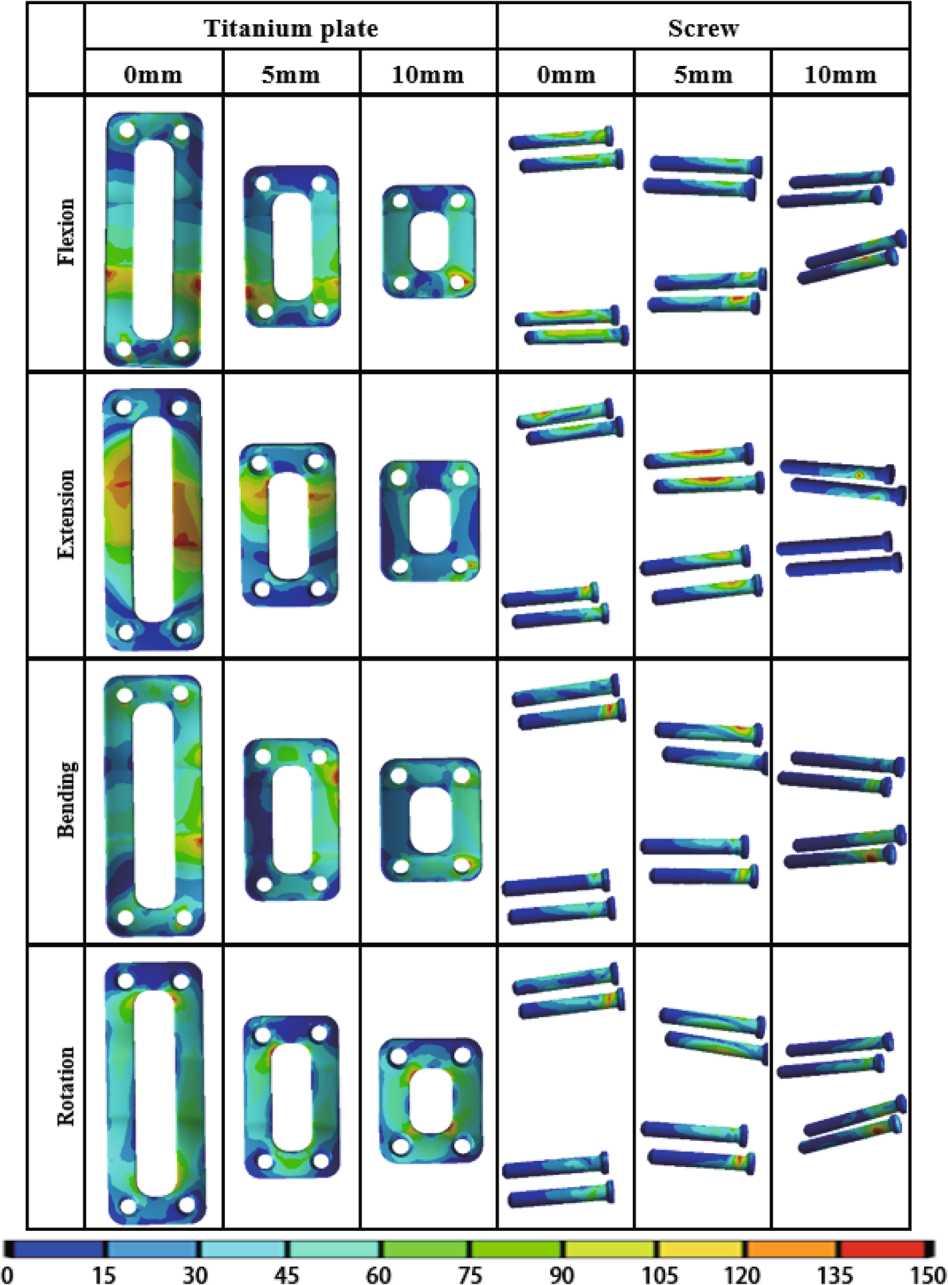
The von Mises stress distribution diagram of titanium plate and screw with different PDD titanium plates under different loading conditions. *PDD* plate-to-disc distance

The von Mises stress of adjacent intervertebral disc, bone graft, titanium plate and screw under different load in different PDD titanium plate groups are shown in Table 6. The stress cloud diagrams are shown in Figs. 5, 6 and 7. No stress concentration point is found in the figures, indicating that the calculation results are reliable.

**Table 6:** The von Mises stress of the structures in different lengths of titanium plates

von Mises stress (MPa)		PDD = 0 mm	PDD = 5 mm	PDD = 10 mm	F	*P*	η^2^
Flexion	C4/C5 disc	1.417 ± 0.297	1.411 ± 0.257	1.422 ± 0.271	0.406	0.671	0.029
	C6/C7 disc	1.483 ± 0.036	1.476 ± 0.045	1.455 ± 0.030	1.495	0.242	0.100
	Bone graft	0.255 ± 0.040 ^* #^	0.307 ± 0.025 ^#^	0.400 ± 0.031	49.897	< 0.001	0.787
	Titanium plate	57.886 ± 3.374 ^* #^	48.363 ± 4.237 ^#^	36.822 ± 1.049	109.670	< 0.001	0.890
	Screw	32.983 ± 1.238 ^* #^	42.075 ± 3.569 ^#^	58.018 ± 3.083	202.663	< 0.001	0.938
Extension	C4/C5 disc	1.337 ± 0.027	1.333 ± 0.019	1.346 ± 0.032	0.619	0.546	0.044
	C6/C7 disc	1.425 ± 0.027	1.419 ± 0.016	1.413 ± 0.018	0.751	0.482	0.053
	Bone graft	1.527 ± 0.051 ^* #^	1.668 ± 0.042 ^#^	1.812 ± 0.053	84.109	< 0.001	0.862
	Titanium plate	58.069 ± 6.173 ^* #^	41.552 ± 3.822 ^#^	24.791 ± 1.740	148.985	< 0.001	0.917
	Screw	34.820 ± 1.386 ^* #^	58.166 ± 3.206 ^#^	95.603 ± 2.204	1653.432	< 0.001	0.992
Bending	C4/C5 disc	1.382 ± 0.038	1.426 ± 0.119	1.388 ± 0.041	0.957	0.397	0.066
	C6/C7 disc	1.402 ± 0.030	1.415 ± 0.032	1.398 ± 0.020	0.960	0.395	0.066
	Bone graft	1.522 ± 0.084 ^* #^	1.868 ± 0.057 ^#^	2.308 ± 0.055	349.902	< 0.001	0.963
	Titanium plate	94.691 ± 1.859 ^* #^	83.956 ± 2.769 ^#^	73.790 ± 1.708	233.352	< 0.001	0.945
	Screw	55.388 ± 1.090 ^* #^	60.705 ± 1.258 ^#^	66.406 ± 0.647	285.569	< 0.001	0.955
Rotation	C4/C5 disc	1.384 ± 0.047	1.378 ± 0.036	1.387 ± 0.038	0.112	0.895	0.008
	C6/C7 disc	1.351 ± 0.170	1.292 ± 0.170	1.224 ± 0.080	1.912	0.167	0.124
	Bone graft	1.620 ± 0.026 ^* #^	1.714 ± 0.053 ^#^	1.945 ± 0.041	161.710	< 0.001	0.923
	Titanium plate	68.463 ± 2.529 ^* #^	62.311 ± 2.526 ^#^	57.824 ± 0.929	62.751	< 0.001	0.823
	Screw	56.617 ± 1.467 ^* #^	64.038 ± 1.663 ^#^	75.025 ± 2.727	208.241	< 0.001	0.939

*PDD* Plate-to-disc distance

^*^ *P<*0.05:Compared with titanium plates of 5 mm PDD

^#^ *P<*0.05:Compared with titanium plates of 10 mm PDD

#### The von Mises stress of adjacent intervertebral disc

The von Mises stress distribution diagram of adjacent intervertebral discs with different PDD titanium plates in different loading conditions are shown in Fig. 5. It can be seen that the PDD had little effect on adjacent intervertebral disc stress distribution. The stress of the nucleus pulposus is smaller than the annulus fibrosus, which may be due to the fact that the stiffness of the annulus fibrosus is greater than that of the nucleus pulposus, so the annulus fibrosus carries more stress. In Table 6, for the upper adjacent segment (C4/C5), no significant differences were found in the von Mises stress of adjacent intervertebral disc among the three different PDD groups in flexion, extension, axial rotation, and lateral bending loading conditions (*P* > 0.05). Additionally, for the lower adjacent segment (C6/C7), there was also no statistical difference among the three groups in different loading conditions (*P* > 0.05).

#### The von Mises stress of bone graft

The von Mises stress distribution diagram of bone graft with different PDD titanium plates in different loading conditions are shown in Fig. 6. The stress of the bone graft varies with the direction of movement, mainly concentrated on the edge of the bone graft, indicating that it has played a good supporting role. It is obvious that the red stress area of the bone graft gradually decreases as the length of the titanium plate increases. In Table 6, the von Mises stress of bone graft in the three groups were statistically different (*P* <  0.05). The bone graft stress decreased as the PDD decreased in all different loading conditions.

#### The von Mises stress of titanium plate

The von Mises stress distribution diagram of titanium plate with different PDD titanium plates in different loading conditions are shown in Fig. 7. The red stress area of the titanium plate gradually increases as the length of the titanium plate increases. In Table 6,a significant difference was found in the von Mises stress of titanium plates among the three different PDD groups (*P* <  0.05). The stress of titanium plate increased as the PDD decreased in all different loading conditions.

#### The von Mises stress of screw

The von Mises stress distribution diagram of screws with different PDD titanium plates in different loading conditions are shown in Fig. 7. Although the area of the red stress does not significantly decrease with the increase of the length of the titanium plate, the maximum stress decreases with the increase of the length of the titanium plate and the stress is mainly concentrated on the two screws at the cranial side. In Table 6, a significant difference in the von Mises stress of screws was found in the three different PDD groups (*P* <  0.05). The screw stress decreased as the PDD decreased in all different loading conditions.

## Discussion

ASD as a complication of ACDF has been reported to cause 17.4% of ACDF patients to have to undergo a second surgery [17], which has seriously affected patient’s quality of life and increased the economic burden on families and society.

Various scholars have different opinions regarding PDD and ASD in ACDF. Chung et al. [14] reported that 177 patients who underwent anterior cervical discectomy and fusion using cervical plates, with follow-up periods of at least 10 years. They found most clinical ASD appeared on the patients with a PDD less than 5 mm. So they considered that to prevent ASD, the PDD should be 5 mm or more if possible. In addition, Yu et al. [20] agreed that PDD < 5 mm is a risk factor for ASD by the logistic regression analysis based on 138 patients. However, Yang et al. [21] retrospectively reviewed 218 patients who underwent anterior cervical arthrodesis with plating and considered that there is no correlation between PDD and the incidence of ASD, but PDD > 5 mm could avoid the development of adjacent segment ossification. Therefore, it remains controversial whether different PDD plate will affect the incidence of ASD.

FEA have been widely used for a biomechanical analysis of the cervical spine because they can analyze various results quantitatively without any invasion. In the present study, we constructed 10 three-dimensional finite element C4-C7 models based on cervical CT images of 10 volunteers. Considering that cervical disc lesions mostly occur in the C5/C6 segments, we selected the C5/C6 segment as the surgical segment so that the results could be suitable for more patients.

In our study, no significant differences were found in the adjacent intervertebral disc stress in the three different PDD groups. This result indicates that PDD does not affect the adjacent intervertebral disc stress. It has been reported that excessive loading can induce degeneration of intervertebral discs. So, we can conclude that the titanium plates of different PDD will not promote the degeneration of adjacent segments by increasing the intervertebral disc stress of adjacent segments. This can further validate the clinical findings of Yang et al. [21] that there is no correlation between PDD and the incidence of ASD.

As for the bone graft stress, we found that the bone graft stress decreased as the PDD decreased. This may be related to the fact that the short PDD plate increases the stiffness of the surgical segment and provides better stability. Although the most appropriate stress on bone graft is not clearly, excessive stress on the bone graft may result in fusion failure because of graft dislodgement and endplate fracture [33]. Furthermore, non-fusion is an important reason for the failure of instruments [34]. Considering bone graft stress and fusion rate, shorter PDD plate is safer because that shorter PDD is helpful to prevent bone graft subsidence and instrument failure from a biomechanical point of view.

The stress of titanium plate increased as the PDD decreased, which shows that as the length of the plate increases, the stress increases accordingly. This is due to the longer torque of the long plate. Of course, this result also explains that long titanium plates can carry more stress, which provides better stability. Although the increase of stress of the plate may cause the plate to break, the increase of the plate stress among different PDD groups is not obvious in the average value.

The screw stress decreased as the PDD decreased. As the length of the steel plate increases, the screw stress decreases. This may be caused by that the most of the overall stress is mostly carried by the plate. Screw loosening and breakage are associated with metal fatigue via pseudarthrosis, which were most dangerous complications in cervical anterior plating fixation. So shorter PDD plate plays an important role in preventing screws breakage and loosening. Yang et al. [21] found when the end point of plate violates the adjacent space, the incidence of ASD is the lowest among different PDD groups. They considered that this may be because the formation of ossification improves the stability of adjacent segments. However, they do not advocate the use of plates that can invade adjacent space. Considering that the strength of the screw is weaker than that of the plate [34], we believe that choosing a slightly shorter PDD plate is more conducive to postoperative recovery. Of course, we also don’t recommend that the PDD is so small that violate the adjacent space.

In addition, we find that the maximum stress of each part occurred was mostly in the conditions of rotation and lateral bending, so we recommend that patients should avoid excessive rotation and lateral bending during early postoperative period to prevent screws breakage, fusion failure, endplate fracture and other complications.

In this study, all materials used linear assignment except for ligaments. Obviously, the material properties of the cervical spine are complex. Structures such as intervertebral discs and annulus are non-linear structures and have viscous properties. Although the nonlinear analysis would probably predict the stress and strain within involved structures in a more realistic situation, the linear parameters can also simulate the real situation well in the case of small torque and small deformation. Hence, we used 1 N·m - a small torque to simulate movement in all directions to ensure that the calculation results were closer to the real data. Model built with too many nonlinearities and complex parameters would greatly increase the non-convergence of calculations, so we assign the various structures of the cervical spine linear parameters. The nonlinear properties of ligaments are very important for the study of spinal biomechanics, so we assigned the ligaments non-parametric properties.

The present study has a number of limitations. Muscles and other soft tissue were not constructed in the models, however, these structures are extremely important for spine biomechanics research. In addition, the screws were designed as solid cylinders bound to the cage or plate, and the threads on the screws were not modeled. Some simplifications were carried out in the prosthesis geometry, for example, we simplify the cancellous bone as a solid structure which may affect the distribution and geometric deformation of the load. These limitations lead to the models cannot completely represent actual in vivo conditions after surgery. Hence, the results from these models should be interpreted carefully. This study aims to reflect a tendency rather than actual data and other situations should be considered in future studies. Nevertheless, to our knowledge, few studies of FEA in cervical spine were based on ten or more models. Compared with only one model, ten models in this study could enhance the accuracy of the results. In a word, although completely duplicating the result of in vivo studies in FEA was impossible, this study effectively shows the biomechanical differences among different PDD plate groups. Meanwhile, further studies with more accurate simulation method still are needed to explore the effect of different PDD in ACDF.

## Conclusions

The PDD has no effect on adjacent intervertebral discs stress, but it is an important factor that affecting the bone graft stress, titanium plate stress and screw stress after ACDF. Shorter PDD does not affect the incidence of ASD through the increase of the adjacent intervertebral discs stress, but it can provide better stability to reduce stress on screws and bone graft which may be helpful to prevent bone graft subsidence, pseudarthrosis and instrument failure. This can serve as a reference for clinical choice of plate.

## Data Availability

The datasets generated and/or analysed during the current study are not publicly available due to individual privacy but are available from the corresponding author on reasonable request.

## Abbreviations

ACDF: Anterior cervical discectomy and fusion
ALL: Anterior longitudinal ligament
ASD: Adjacent segment degeneration
CL: Capsular ligament
CT: Computed tomography
FE: Finite element
FEA: Finite element analysis
ISL: Interspinous ligament
LF: Ligamentum flavum
PDD: Plate-to-disc distance
PLL: Posterior longitudinal ligament
ROM: The range of motion
